# Transcriptomic signatures for human male infertility

**DOI:** 10.3389/fmolb.2023.1226829

**Published:** 2023-08-21

**Authors:** Alenka Hodžić, Aleš Maver, Branko Zorn, Daniel Petrovič, Tanja Kunej, Borut Peterlin

**Affiliations:** ^1^ Clinical Institute of Genomic Medicine, University Medical Centre Ljubljana, Ljubljana, Slovenia; ^2^ Andrology Unit, Reproductive Unit, Department of Obstetrics and Gynecology, University Medical Centre Ljubljana, Ljubljana, Slovenia; ^3^ Institute of Histology and Embryology, Faculty of Medicine, University of Ljubljana, Ljubljana, Slovenia; ^4^ Biotechnical Faculty, University of Ljubljana, Ljubljana, Slovenia

**Keywords:** idiopathic male infertility, gene expresion, transcriptome, testis, spermatogenesis

## Abstract

**Introduction:** Male infertility is a common, complex disorder. A better understanding of pathogenesis and etiology is needed for timely diagnosis and treatment. The aim of this study, therefore, was to identify genes involved in the pathogenesis of idiopathic male infertility based on data from transcriptomic level supported with data from genomic level.

**Materials and methods:** First, we performed whole gene expression analysis in 20 testis biopsy samples of patients with severely impaired (10) and normal spermatogenesis (10). Further, we have performed systematic review of comparable male infertility studies and overlapped the most significantly expressed genes identified in our study with the most differentially expressed genes from selected studies. Gene Ontology analysis and KEGG functional enrichment have been performed with Enrichr analysis tool. Additionally, we have overlapped these genes with the genes where rare variants have been identified previously.

**Results:** In 10 patients with severely impaired spermatogenesis and 10 controls, we identified more than 1,800 differentially expressed genes (*p* < 0.001). With the systematic review of three previously performed microarray studies that have met inclusion criteria we identified 257 overlapped differentialy expressed genes (144 downregulated and 113 upregulated). Intersection of genes from transcriptomic studies with genes with identified rare variants revealed a total of 7 genes linked with male infertility phenotype (CYP11A1, CYP17A1, RSPH3, TSGA10, AKAP4, CCIN, NDNF).

**Conclusion:** Our comprehensive study highlighted the role of four genes in pathogenesis of male infertility and provided supporting evidence for three promising candidate genes which dysfunction may result in a male infertility disorder.

## 1 Introduction

Male infertility is a worldwide health problem affecting about 7% of adult human males (Krausz and Riera-Escamilla, 2018). Despite the considerable research effort, in a large proportion of infertile males the cause of their infertility is unknown (idiopathic), whereas genetic factors have been considered as a major contributing cause for them (Hwang et al., 2010).

Studies of animal models have identified hundreds of genes that influence reproduction and have uncovered various pathogenic pathways that could affect infertility in humans. Still, only a few mutations in human genes have been associated with male infertility (Jamsai and O’Bryan, 2010; Massart et al., 2011). One probable explanation is that for many years genetic research on male infertility involved the targeted sequencing of individual genes in cohorts of infertile men and fertile controls with a limited number of whole-exome sequencing studies performed in the last few years (Fakhro et al., 2018; Hodžić et al., 2020).

Human spermatogenesis is a complex process; therefore, these targeted approaches proved ineffective in identifying genetic variants responsible for infertility. Global gene-expression profiling has been used extensively in studying complex diseases and have already demonstrated potential for identifying genomic features associated with various diseases (Cookson et al., 2009). Contrary to rodent models and other pathologies in humans, attempts to perform similar investigation in the field of male infertility have been limited due to various restrictions (e.g., restrictions to human testis tissue).

A few microarray studies have been performed previously and have provided valuable insights into the complexity of spermatogenesis by identifying numerous testis expressed and testis-specific genes in humans (Feig et al., 2006; Ellis et al., 2007; Spiess et al., 2007; Okada et al., 2008). However, individual studies are usually not reproducible, and comparing studies with different designs may conceal the specific mechanisms underlying infertility. Kui et al. (2019) integrated results from different microarray data sets and focused on differentially expressed genes enriched in four divided pathological phenotypes (Kui et al., 2019). Furthermore, we hypothesized that integrating the data extracted from the transcriptomic level with the data originating from the genomic level could led us to identify potential biomarkers for male infertility. Namely, like all “omic” approaches, findings of rare, potentially pathogenic variants in infertile men need further evidence to distinguish the real signal from noise.

For this purpose, we performed global gene expression profiling on human testis samples in patients with severely impaired and normal spermatogenesis. In addition to our study, we systematically reviewed overlapping genes from comparable previous studies. We identified genes demonstrating differential expression and rare potentially pathogenic variants as a final step.

## 2 Materials and methods

### 2.1 Ethics statement

The study was approved by National medical ethics committee (reference number: 73/05/12). All patients gave informed written consent to participate in the study.

### 2.2 Testicular biopsy samples

All biopsy samples were obtained from patients attending the outpatient infertility clinic of the Andrology Centre, Department of Obstetrics and Gynecology in Ljubljana. The study population comprised ten patients with non-obstructive azoospermia and ten patients with obstructive azoospermia (controls).

Testicular biopsies were performed under local anaesthesia. Following unilateral hemiscrototomy, a small testicular incision was made and at least two samples of testicular tissue were taken from each testis. The first sample was fixed in Bouin’s solution, routinely embedded in paraffin, and cut at a section thickness of 5 μm. The sections were stained with HE, PAS and van Gieson–Weigert. A systematic histological evaluation was performed under light microscopy. More than 100 seminiferous tubules were scored for each patient. The results were expressed as a relative number of tubules showing Sertoli cells, spermatogonia, spermatocytes, round and elongated spermatids, and spermatozoa. All examinations were made by the same observer (J.S.). The diagnoses were as follows: 10 patients with normal spermatogenesis, 2 with early and late maturation arrest and 8 with Sertoli-cell-only syndrome (SCOS). Before sperm recovery, medical history was established, testicular volume measured and serum FSH level was assessed. The clinical characteristics of patients are presented in Table 1.

**TABLE 1:** Clinical characteristics of patients included in the transcriptome profiling study.

Patient	Clinical findings^a^	Testicular volume (mL)	FSH level (IU/l)	Sperm count^a^	Sperm motility^b^	Histology^b^	Johnsen’s score
1	bilateral epididymal enlargement	12/13	3,7	3	2	normal spermatogenesis	10
2	bilateral epididymal enlargement	25/28	4,75	3	2	normal spermatogenesis	9
3	bilateral epididymal enlargement	13/16	3,2	3	2	normal spermatogenesis	9
4	bilateral epididymal enlargement	22/25	7,02	3	2	normal spermatogenesis	9
5	CABVD	14/14	3,63	3	2	normal spermatogenesis	9
6	CABVD	28/28	1,68	2	0	normal spermatogenesis	9
7	enlarged right epididymis	15/15	8,3	3	2	Hypospermatogenesis, mononuclear infiltration	9
8	enlarged right epididymis 8 years after	15/15	2,2	3	2	hypospermatogenesis	9
9	no enlargement	25/25	1,79	2	1	hypospermatogenesis	9
10	enlarged right epididymis	30/30	1,27	2	2	hypospermatogenesis, EMA	9
11	NA	NA	NA	0	0	LMA	6
12	no	15/15	12,5	0	0	SCOS, EMA, hypospermatogenesis	5
13	no	20/20	14,2	0	0	SCOS, EMA	5
14	no	12/15	12,8	0	0	SCOS, EMA	5
15	no	8/10	13	0	0	SCOS, EMA	5
16	hypogonadism	1/1	82	0	0	SCOS, EMA	2
17	hypogonadism	6/4	59,8	0	0	SCOS, LCH	2
18	no	15/15	44	0	0	SCOS, EMA	2
19		8/10	28,7	0	0	SCOS, EMA	1
20	hypogonadism	2/2	36,4	0	0	SCOS, LCH	1

^a^ CABVD, congenital bilateral absence of vasa deferentia.

^b^ EMA, early maturation arrest; LMA, late maturation arrest; LCH, Leydig cell hyperplasia; NA, not available.

a0 = no sperm; 1 = rare; 2 = low; 3 = numerous. b0 = immotile; 1 = non-progressively motile; 2 = progressively motile.

### 2.3 RNA isolation

Total RNA was isolated from frozen testis biopsy samples by using the TissueLyserLT (Qiagen) apparatus and RNeasy Plus Micro kit (Qiagen) according to the manufacturer’s instructions. About 3.5–4.5 mg weight of tissue was disrupted and homogenized in 2 mL centrifuge tubes containing 5-mm stainless steel beads and lysis buffer (Buffer RLT plus) at a frequency of 50 Hz for 10 min in a TissueLyser device. Homogenate was subsequently centrifuged for 3 min, collected and eluted in a gDNA eliminator spin column. Ethanol was added to the flow-through to provide appropriate binding conditions for RNA and samples were then applied to an RNeasy MinElute spin columns, where total RNA was bound to the membrane and contaminants were efficiently whashed away. RNA was then eluted in 14 µL of water. RNA concentrations were determined using NanoDrop 2000C Spectrophotometer (Thermo Scientific) and RNA quality was verified by Agilent Bioanalyzer 2,100 (Agilent Technologies).

### 2.4 Microarray experiment and data analysis

The measure of expression was performed on isolated RNA from biopsy samples of patients with obstructive and non-obstructive azoospermia using Agilent Whole Human Genome 4 × 44 microarrays (Agilent design id: 14850). Gene expression platform contains 264 × 10 biological probes, targeting altogether 19.596 unique mRNA sequences according to NCBI Reference Human Genome Build version 33. Sample preparing, labeling and amplification RNA (Low Input Quick Amp Labeling Kit, two color, Agilent Technologies), hybridization, washing and scanning were performed according to the manufacturer’s recommendations (Agilent Technologies). Differential gene expression was measured in relation to the Agilent’s Universal Human Reference RNA and each experimental sample was hybridized against this common reference sample.

After hybridization, microarray slides were scanned using Agilent High Resolution Microarray Scanner System, using the manufacturer’s recommended scanning settings. Subsequently, microarray features were extracted using Agilent Feature Extraction software v10.7.3.1. Post-processing steps included intra-array lowess and inter-array quantile normalization to correct for potential bias resulting from differential stability of cyanine dyes. Fluorescent values were offset by 100 units to reduce the anomalous dispersion of fold change (FC) values at lower signal intensities. MA and multidimensional scaling (MDS) plots were inspected for each array to detect any systemic error resulting from the preceding steps.

The raw data are deposited in NCBI’s Gene Expression Omnibus (Edgar et al., 2002) and are accessible through GEO Series accession number GSE145467 (https://www.ncbi.nlm.nih.gov/geo/query/acc.cgi?acc=GSE145467).

Results were statistically analyzed using a linear model fit in limma package for Bioconductor in an R statistical environment. To account for multiple testing, obtained significance values were corrected using the Benjamini-Hochberg method, and the adjusted significance threshold was set at <0.05.

### 2.5 Systematic review

To identify overlapping genes from studies that could be included in the systematic review, a PubMed search (www.ncbi.nlm.nih.gov/pubmed/) until June 2022 was performed using the search strings: ‘male infertility’ AND ‘human spermatogenesis’ AND ‘gene expression’ AND ‘testis’. The systematic review was conducted according to the Preferred Reporting Items for Systematic Reviews and Meta-analyses (PRISMA) statement (Moher et al., 2009). The following criteria were used: (a) comparable disease states as in our experiment, (b) the same tissue samples, (c) common reference design approach towards measuring differential expression in the microarray experiment and (d) availability of raw datasets at either GEO or ArrayExpress repositories.

All the steps described in the following sections were performed in using R statistical language version 2.9.2, using Bioconductor environment.

Due to the differences in methodology and statistical analyses among the 3 studies, FDR values were not used, and instead, differentially expressed transcripts with *p* < 0.001, were used for the comparisons. Shared differentially expressed genes were visualized using an online VENN tool, available from the VIB/UGent Bioinformatics & Systems Biology (http://bioinformatics.psb.ugent.be/cgi-bin/liste/Venn/calculate_venn.htpl).

To gain more insight into key processes that may possibly explain functional differences among the testicular samples from severely impaired and normal spermatogenesis types, we carried out functional annotation analysis of the top overlapping differentially expressed genes, attaining adjusted *p*-values below 0.001 and using hypergeometric test, based on genes’ relation to GeneOntology (GO Biological Process 2021; https://maayanlab.cloud/Enrichr/) and KEGG terms (KEGG 2021 Human, https://maayanlab.cloud/Enrichr/).

### 2.6 Common genes identified in both transcriptomic and sequencing studies searching for monogenic etiology of male infertility

To provide additional support for identified genes from transcriptomic studies, we searched for overlapped differentially expressed genes with evidence to be putative candidates for the monogenic aetiology of male infertility. For this purpose, we have used the selected genes from a systematic review of next-generation sequencing studies, identifying rare, potentially pathogenic variants published recently (Houston et al., 2021). We have included genes moderately, strongly, or definitively linked to male infertility phenotypes and genes with limited evidence. We have excluded genes scored as “no evidence”.

## 3 Results

### 3.1 Global gene-expression profiling

Using Agilent Whole Human Genome 4 × 44 microarrays (Agilent design id: 14850), we analyzed global gene expression changes in testis biopsy samples from 10 subjects with severely impaired spermatogenesis and 10 with normal spermatogenesis. Excluding one sample identified as an outlier left 19 samples for analysis. With a strict statistical criterion (adj. p < 0.001), we identified more than 1801 differentially expressed genes. Of these 1801, 781 were downregulated and 1,020 were up-regulated.

### 3.2 Systematic review and gene set enrichment analyses

Among the identified 628 articles, transcriptomic profiling of the human testis was identified in 10 articles. Of these 10 articles, only three previously performed transcriptomic studies have met inclusion criteria. Therefore GEO datasets with accession numbers GSE9210, GSE4797 and GSE45885, in addition to our own microarray results, were finally included in the analysis. Details on the three studies included in the systematic review are given in Table 2.

**TABLE 2:** Description of datasets included in the meta-analysis.

Dataset accession	Number of samples—azoospermia	Number of samples—obstructive forms of infertility	Array platform	Number of probes on the array
GSE145467 (our study)	10	10	Agilent-014850 Whole Human Genome Microarray 4 × 44K G4112F	∼19.596 probes
GSE9210	47	11	Agilent-012097 Human 1A Microarray (V2) G4110B	∼22000 probes
GSE4797	5	18	GE Healthcare/Amersham Biosciences CodeLink™ UniSet Human 20K I Bioarray	∼23000 probes
GSE45885	27	4	Affymetrix Human Gene 1.0 ST Array	∼28132 probes

**FIGURE 1:**
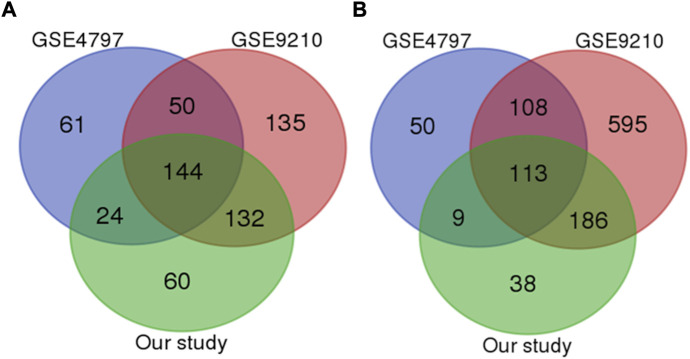
Venn diagram of the most significant downregulated **(A)** and upregulated genes **(B)** genes among all studies (*p* value less than 0.0001).

Aiming to identify the specific mechanisms underlying infertility, we focused on overlapping genes among four analyzed studies. With a strict statistical criterion (*p* < 0.001), we successfully identified a signature comprised of 257 shared genes (144 down-regulated and 113 up-regulated) with changed expression in infertile men (Figures 1A, B). However, one study did not successfully pass through this sorting criterion. Considering good overlap between the other three studies, the possible reason for this deviation could be in the small set of control samples.

Top biological processes according to GO enriched with common genes across all studies were genes related to collagen fibril organization and spindle and mitotic assembly checkpoint signaling, while according to KEGG pathways, common genes across all studies were genes related to ovarian steroidogenesis and p53 signaling pathway (Bar Chart 1 and Bar Chart 2).

**BAR GRAPH 1:**
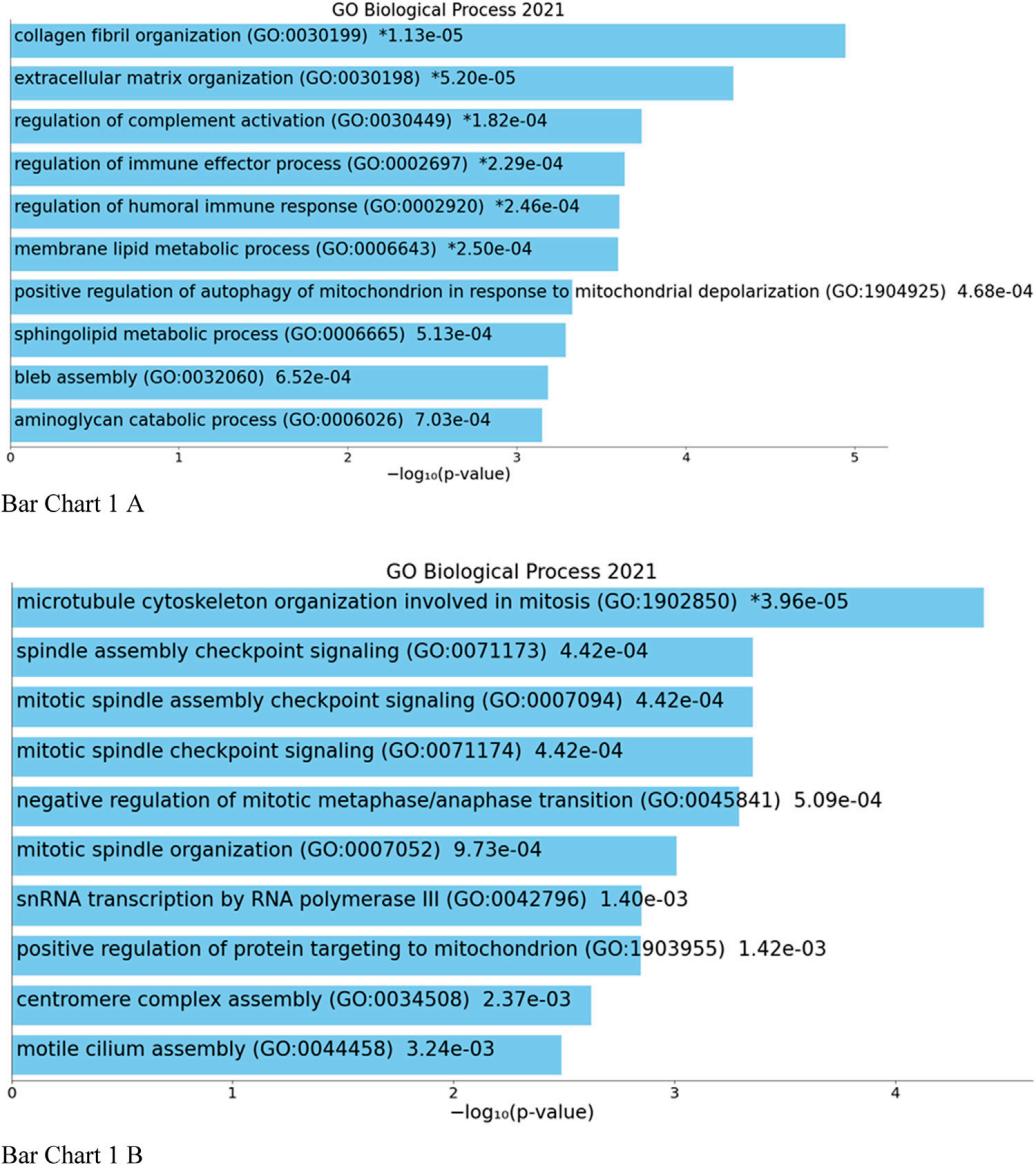
Gene set enrichment analysis of common down-regulated and up-regulated genes across all four studies using GENE ONTOLOGY (biological process) [**(A)** up-regulated, **(B)** down-regulated].

**BAR GRAPH 2:**
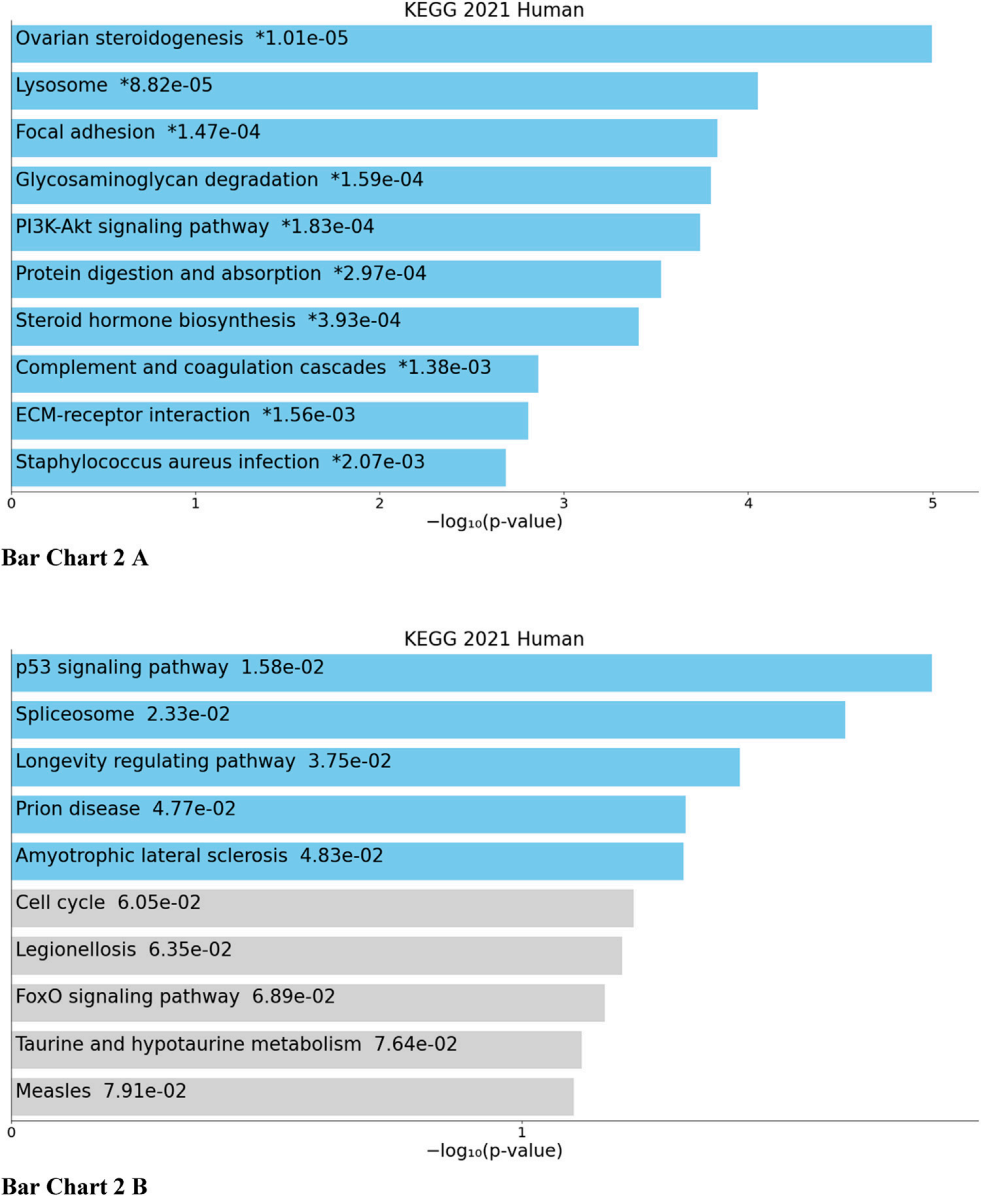
Gene set enrichment analysis of common down-regulated and up-regulated genes across all four studies using KEGG pathway functional annotation [**(A)** up-regulated, **(B)** down-regulated].

### 3.3 Common genes identified in transcriptomic and next-generation sequencing studies

The intersection of genes from transcriptomic studies and those reported as candidates for monogenic aetiology revealed seven genes linked with male infertility phenotype. CYP11A1, CYP17A1, RSPH3, and TSGA10 genes were classified as confidently associated with the phenotype (moderate, strong, or definitive), whereas AKAP4, CCIN, NDNF present genes classified as limited evidence.

## 4 Disscusion

In the present study, we performed global gene expression profiling on human testis samples in patients with severely impaired spermatogenesis and systematically reviewed previous transcriptomic studies to identify genes consistently differently expressed across the studies.

To provide additional support, we searched for differentially expressed genes with evidence to be putative candidates for the monogenic aetiology of male infertility. The integratomic approach revealed four genes classified as confidently causally associated with phenotype, whereas another three present candidate genes whose dysfunction may result in a male infertility disorder.

CYP11A1, CYP17A1 and RSPH3 were upregulated in patients with normal spermatogenesis when compared to the patients with impaired spermatogenesis.

Considering that CYP17A1 and CYP11A1 are required for proper synthesis of androgens, noticed upregulation in patients with impaired spermatogenesis could result from organism efforts to maintain stability. Biallelic variants in CYP11A1 and CYP17A1 gene can impair sexual differentiation caused by a complete or partial loss of steroid hormone production. The broad clinical spectrum has been reported in affected 46XY males and may vary from normal genitalia and surgically repairable defects, including cryptorchidism and hypospadias, to complete feminization of external gonads, accompanied by adrenal dysfunction (Sherbet et al., 2003; Rubtsov et al., 2009; Kok et al., 2010; Parajes et al., 2012; Lara-Velazquez et al., 2017; Kolli et al., 2019; Kallali et al., 2020). The broad phenotypic spectrum has been reported for numerous genes, and in this manner, establishing a specific genotype-phenotype relationship is not necessarily straightforward. Variability in genotype-phenotype correlation have been previously recognized in reproductive system syndromes with endocrine disorders. One such example is the NR5A1 gene, where pathogenic variants are associated with a phenotypic spectrum ranging from disorders of sex development to oligo/azoospermia (Domenice et al., 2016).

RSPH3 biallelic variants represent a recurrent genetic cause of Primary ciliary dyskinesia (PCD) in human (Jeanson et al., 2015). Although the RSPH3 gene is abundantly expressed in testicular tissue, the phenotype of PCD-associated male infertility caused by the defect in this ciliopathy-related gene has been rarely described (Wu et al., 2020). Mutations in RSPH3 have been reported in a sterile PCD patient with severe asthenoteratospermia characterized by multiple flagellar malformations (Wu et al., 2020). However, the mutation in this gene in association with non-obstructive azoospermia has not been identified so far.

Based on our analyses, the expression levels of the TSGA10 gene were downregulated across studies. It has been suggested that decreased expression of TSGA10 is associated with the reduction of autophagy process and increased ROS levels, which lead to aberrant spermatid differentiation and maturation (Asgari et al., 2021). The TSGA10 gene is involved in active cell division, differentiation and cell migration and is highly conserved among different species (Behnam et al., 2009). Functional analysis of the Tsga10 gene in knockout mice demonstrated that TSGA10 contribute to the correct arrangement of a mitochondrial sheath in spermatozoa, and its deficiency leads to male infertility (Luo et al., 2020). Previous studies have reported a homozygous mutation in this gene in patients with acephalic spermatozoa (Sha et al., 2018; Ye et al., 2020).

According to the literature, there has been limited evidence for the association of AKAP4, CCIN and NDNF genes with male infertility. Two recently published studies identified a homozygous missense mutation and a compound heterozygous mutation of the CCIN gene in patients with teratozoospermia, while a missense variant in the AKAP4 gene was associated with morphological abnormalities of the sperm flagella phenotype (He et al., 2023; Fan et al., 2022; Zhang et al., 2021). Both genes were significantly downregulated in patients with impaired spermatogenesis across included studies, making them interesting candidates for future research of male infertility.

## 5 Conclusion

In the first step, we identified 257 differentially expressed genes, which are according to KEGG, mainly involved in p53 signaling and steroidogenesis pathways. In the second step we identified seven genes demonstrating both differential expression and evidence of rare, putative pathologic genetic variants (CYP11A1,CYP17A1,RSPH3,TSGA10,AKAP4,CCIN, NDNF). With the integratomic approach, we contributed to building evidence for genetic etiology and mechanisms in male infertility.

## Data Availability

The datasets presented in this study can be found in online repositories. The names of the repository/repositories and accession number(s) can be found in the article/Supplementary Material.
